# Multi-scale spatial heterogeneity of pectic rhamnogalacturonan I (RG–I) structural features in tobacco seed endosperm cell walls

**DOI:** 10.1111/tpj.12263

**Published:** 2013-06-21

**Authors:** Kieran JD Lee, Valérie Cornuault, Iain W Manfield, Marie-Christine Ralet, J Paul Knox

**Affiliations:** 1 Centre for Plant Sciences, Faculty of Biological Sciences, University of LeedsLeeds, LS2 9JT, UK; 2 Astbury Centre for Structural Molecular Biology, Faculty of Biological Sciences, University of LeedsLeeds, LS2 9JT, UK; 3 UR1268 Biopolymères, Interactions et Assemblages, Institut National de la Recherche AgronomiqueRue de la Géraudière, BP 71627, F–44316, Nantes, France

**Keywords:** Nicotiana tabacum, seed endosperm, cell wall, rhamnogalacturonan-I, pectin

## Abstract

Plant cell walls are complex configurations of polysaccharides that fulfil a diversity of roles during plant growth and development. They also provide sets of biomaterials that are widely exploited in food, fibre and fuel applications. The pectic polysaccharides, which comprise approximately a third of primary cell walls, form complex supramolecular structures with distinct glycan domains. Rhamnogalacturonan I (RG–I) is a highly structurally heterogeneous branched glycan domain within the pectic supramolecule that contains rhamnogalacturonan, arabinan and galactan as structural elements. Heterogeneous RG–I polymers are implicated in generating the mechanical properties of cell walls during cell development and plant growth, but are poorly understood in architectural, biochemical and functional terms. Using specific monoclonal antibodies to the three major RG–I structural elements (arabinan, galactan and the rhamnogalacturonan backbone) for *in situ* analyses and chromatographic detection analyses, the relative occurrences of RG–I structures were studied within a single tissue: the tobacco seed endosperm. The analyses indicate that the features of the RG–I polymer display spatial heterogeneity at the level of the tissue and the level of single cell walls, and also heterogeneity at the biochemical level. This work has implications for understanding RG–I glycan complexity in the context of cell-wall architectures and in relation to cell-wall functions in cell and tissue development.

## Introduction

Plant cell walls have fundamental roles in the growth of plants, and their structures affect cell and organ robustness, cell extension and mechanics, and also function in plant responses to mechanical stress. Chemical understanding of cell walls, in terms of their component polymers, is well advanced, but the biology of cell-wall polysaccharides is less so, and it is not yet understood how distinct cell-wall properties are generated in different cell types or in response to environmental changes (Baskin, 2005; Thompson, 2005; Sarkar *et al*., 2009; Burton *et al*., 2010). Cell walls are complex composites of polysaccharides with lower levels of proteins and other molecules. All land plant cell walls comprise sets of polysaccharides that include cellulose in the form of microfibrils and a range of non-cellulosic polysaccharides. These non-cellulosic polymers include the pectic polysaccharides (defined by the presence of abundant galacturonic acid) which may be interlinked in extensive supramolecules within cell-wall matrices (Caffall and Mohnen, 2009; Burton *et al*., 2010; Yapo, 2011a,b). Other non-pectic and non-cellulosic polysaccharides, sometimes classed as hemicelluloses, include xyloglucans, heteroxylans, heteromannans and mixed-linkage glucans, which vary in distribution and abundance taxonomically and have been proposed to link cellulose microfibrils to form load-bearing networks (Burton *et al*., 2010; Scheller and Ulvskov, 2010).

The complex set of pectic polymers comprises a range of distinct glycan domains. The most abundant and well characterized of these is homogalacturonan (HG), consisting of chains of 1,4–linked GalA residues that are extensively methyl-esterified during synthesis and de-esterified *in muro* by pectin methylesterases. This structural modulation within the wall has the capacity to locally induce cation-based cross-linking, which influences cell-wall porosity and capacity for cell extension (Micheli, 2001; Wolf *et al*., 2009). A structurally conserved, HG-related, extreme heterogeneity, designated rhamnogalacturonan II (RG–II), is a distinct domain of HG with complex heteroglycan side branches that is conserved across species and may be cross-linked by boron. RG–II has been proposed to influence the links and integrity of HG chains and hence cell walls (Pérez *et al*., 2003; O'Neill *et al*., 2004).

A heterogeneous set of branched polymers known as rhamnogalacturonan I (RG–I) is structurally distinct from RG–II (Ridley *et al*., 2001; Willats *et al*., 2001; Caffall and Mohnen, 2009; Yapo, 2011b). RG–I may account for up to 50% of pectic molecules. The rhamnogalacturonan backbone is decorated with glycan side chains, of which 1,5–arabinan and 1,4–galactan are the major structural features among a range of variably present arabinosyl- and galactosyl-rich structures (Ridley *et al*., 2001; Willats *et al*., 2001; Caffall and Mohnen, 2009; Yapo, 2011b). RG–I polymers appear to be widely distributed amongst plants, and both physicochemical and immunocytochemical analyses indicate extensive heterogeneity, with, for example, arabinan- or galactan-rich forms dominant in certain tissues or organs and at different stages of cell development (Willats *et al*., 2001; Caffall and Mohnen, 2009; Verhertbruggen *et al*., 2009b; Yapo, 2011b; Lee *et al*., 2012). A range of studies have implicated RG–I in the generation of cell-wall properties. The trimming of galactosyl side chains of the RG–I found in seed coat mucilage is important for its correct hydration properties (Dean *et al*., 2007; Macquet *et al*., 2007). RG–I has been implicated in cell-wall biomechanical properties such as extensibility and firmness. Evidence indicates that galactan-rich forms contribute to cell-wall firmness or stiffness and arabinan-rich forms contribute to relative cell-wall flexibility or elasticity (McCartney *et al*., 2000; Jones *et al*., 2003; Ulvskov *et al*., 2005; Moore *et al*., 2008, 2013). Genetic interventions leading to enzymatic disruption of RG–I structures indicated that RG–I polymers are important for meristem and developmental functions, but the mechanistic details are not fully understood (Oomen *et al*., 2002; Skjøt *et al*., 2002). In the case of the Arabidopsis root, cell walls at the apical region appear to be arabinan-rich, and a transient occurrence of galactan-rich cell walls marks the switch to cell elongation (McCartney *et al*., 2003; Talboys *et al*., 2011). How the occurrence of the RG–I epitopes in these cases relates to the inter-relationships between the underlying RG–I biochemistry, cell-wall properties and developmental cell status/dynamics is not known.

How to develop understanding of RG–I structure–function relationships within meristems and developing tissue systems remains a challenging question. To understand the cellular functions of the RG–I glycan system and its functional context within pectic supramolecules, it is important to increase knowledge of how RG–I structural elements are integrated within cell walls at the levels of cells, tissues and organs. Here we report dissection of the arabinan, galactan and RG backbone structural elements of RG–I in relation to cell walls within a single tissue: the endosperm of tobacco seeds. Recent work has indicated that tobacco seed endosperm is heteromannan-rich rather than cellulose-rich, and that it has spatial heterogeneity in relation to the micropylar endosperm (ME) and chalazal/peripheral endosperm (collectively referred to as the non-micropylar endosperm; NME) (Lee *et al*., 2012). Here we present detailed *in situ* analysis of RG–I epitopes (LM5 galactan, LM6 arabinan and the INRA-RU1 rhamnogalacturonan backbone) at the level of tissues and single cell walls. This analysis is combined with an approach to assess the underlying biochemical complexity of RG–I polymers by use of molecular probes as detection tools for chromatographic separation of RG–I polymers. Understanding RG–I complexity within a single tissue provides insight into RG–I structures in relation to cell walls and into the structure–function relationships of RG–I polymers in plant cell and tissue development.

## Results

### Heterogeneity in detection of RG–I structural features at the tissue level

#### The LM6 arabinan epitope is detected non-uniformly in tobacco seed endosperm, reflecting a cell-wall asymmetry that is also indicated by Calcofluor White binding

**Figure 1:**
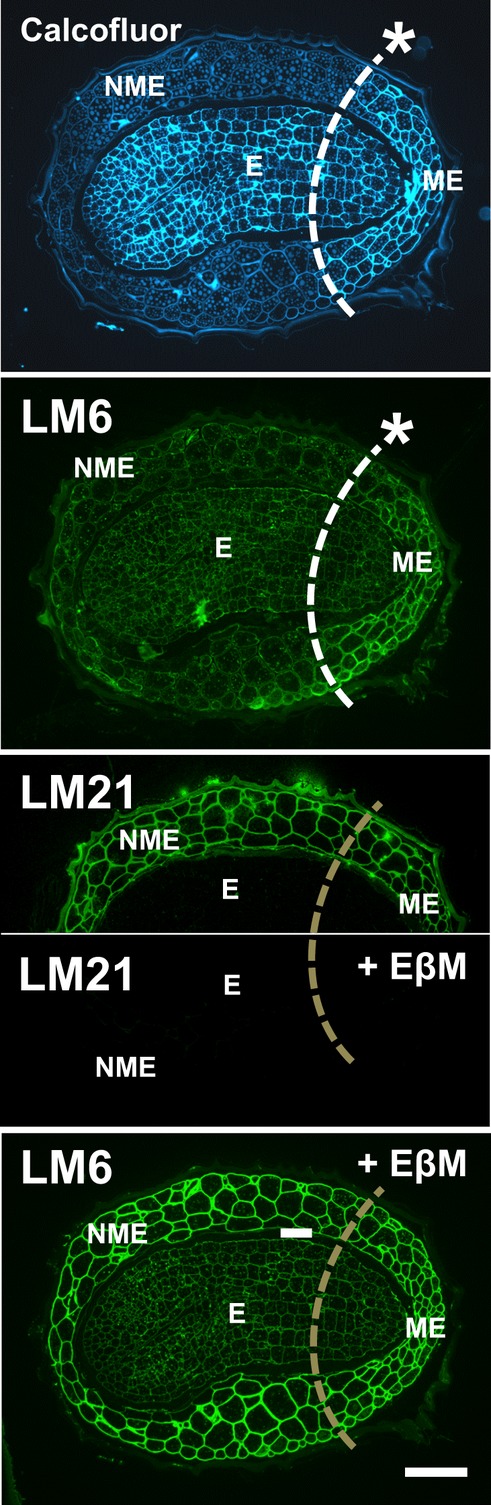
Indirect immunofluorescence labelling of sections through tobacco seeds with Calcofluor White, LM6 arabinan and LM21 heteromannan probes. In some cases, the equivalent sections were pre-treated with endo-β–mannanase (+EβM) to remove the abundant heteromannan. E, embryo; ME, micropylar endosperm; NME, non-micropylar endosperm. White dashed lines with an asterisk indicate that a tissue asymmetry is detected. Brown dashed lines indicate no tissue level asymmetry was detected. Scale bar = 50 μm.

Calcofluor White staining of a resin-embedded medial longitudinal section through a 3 h-imbibed tobacco seed reveals strong fluorescence of all embryo cell walls and asymmetry of the fluorescence intensity of cell walls in the endosperm, with stronger fluorescence in cell walls at the ME adjacent to the embryo radicle apex, as shown in Figure 1. This asymmetry within the endosperm tissue, revealed by Calcofluor White, reflects other structural features of tobacco seed endosperm cell walls as described previously (Lee *et al*., 2012). Immunolabelling of an equivalent section with the LM6 monoclonal antibody, which is directed to 1,5–α–arabinan, indicates the presence of the arabinan epitope in all embryo and endosperm cell walls, with relatively greater detection of the epitope in the ME, reflecting the stronger staining by Calcofluor White (Figure 1).

#### Enzymatic removal of abundant heteromannan from endosperm cell walls results in detection of the LM6 arabinan epitope uniformly throughout the endosperm tissue

Recent work has demonstrated that, for *in situ* cell-wall immunochemistry analyses, the presence of an abundant polysaccharide may block the detection of other polysaccharides or mask them (Marcus *et al*., 2008, 2010). Heteromannan is abundant in tobacco seed endosperm cell walls (Lee *et al*., 2012). Its enzymatic removal using an endo-β–mannanase (EβM), as evidenced by loss of binding of the LM21 heteromannan antibody probe (Figure 1), results in uniform detection of the LM6 arabinan epitope in all cell walls of the seed endosperm, with no detectable asymmetry across the tissue reflecting ME and NME regions as shown in Figure 1. This observation indicates that the differential binding of the LM6 probe to intact cell walls with heteromannan present is due to differential masking of the LM6 epitope, possibly reflecting differential cell-wall porosities across the endosperm.

#### Asymmetry in the detection of the LM5 galactan epitope in the seed endosperm is not altered by enzymatic removal of heteromannan

**Figure 2:**
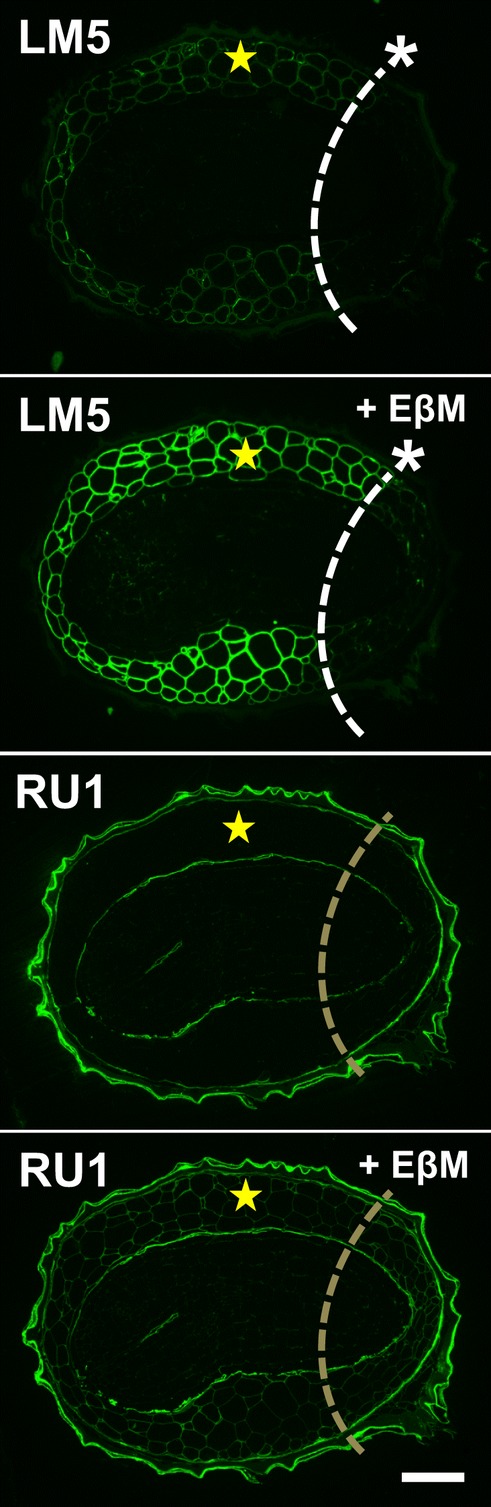
Indirect immunofluorescence labelling of sections through tobacco seeds with LM5 galactan and RU1 rhamnogalacturonan I backbone probes. In the lower micrographs of the micrograph pairs, equivalent sections were treated with with endo-β–mannanase (+EβM) to remove the abundant heteromannan. White dashed lines with an asterisk indicate that a tissue asymmetry was detected. Brown dashed lines indicate no tissue level asymmetry was detected. The yellow stars indicate the region of peripheral endosperm used for fluorescence imaging at higher magnification (see Figure 3). Scale bar = 50 μm.

Immunolabelling of equivalent sections of tobacco seeds with the LM5 galactan monoclonal antibody revealed tissue asymmetry, in that the epitope was detected in the NME but not in the ME region (Figure 2). After enzymatic removal of heteromannan, detection of the LM5 galactan increased in terms of fluorescence intensity, but there was no change in the asymmetry of its occurrence as shown in Figure 2. Further enzymatic deconstructions of endosperm cell walls involving enzymatic removal of pectic HG and pectic arabinan, which are known to be abundant in the ME cell walls (Lee *et al*., 2012), did not result in increased detection of the LM5 galactan epitope in this region.

#### Detection of the INRA-RU1 RG backbone epitope is unmasked by enzymatic removal of heteromannan and is detected uniformly throughout the seed endosperm

Fluorescence imaging of the INRA-RU1 and INRA-RU2 RG backbone epitopes (hereafter RU1 and RU2) in equivalent sections of tobacco seeds indicated that the epitopes were present in the testa cell walls and at the surface of the embryo, but were not present in the endosperm cell walls as shown for RU1 in Figure 2. The RU1 antibody was used for all subsequent *in situ* analyses. Enzymatic removal of the heteromannan resulted in detection of the RU1 epitope in all endosperm cell walls, with no detected asymmetry relating to the ME region.

These observations indicate that the LM5, LM6 and RU1 epitopes, all associated with RG–I, have differential occurrences across the endosperm tissue, with some cell walls having all three epitopes and those of the ME region having the arabinan and RU1 epitopes but a very low level of the galactan epitope. The presence of abundant heteromannan masks access to the RG backbone epitope and results in differential access to the arabinan epitope.

### Heterogeneity in detection of RG–I structural features at the level of single cell walls in the NME

#### Differential occurrence of the RG-related epitopes LM5 galactan, LM6 arabinan and RU1 RG–I backbone within cell walls in the seed endosperm

**Figure 3:**
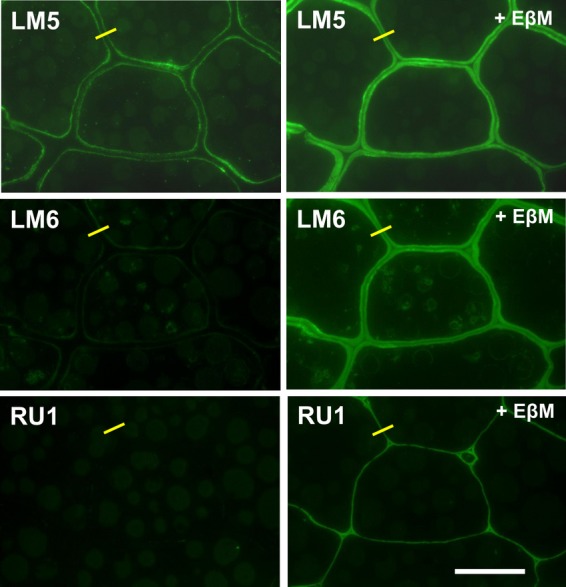
Indirect immunofluorescence labelling of sections through tobacco endosperm cells immuno with LM5 galactan, LM6 arabinan and RU1 RG backbone antibodies using untreated sections (left) and after pre-treatment with endo-β–mannanase (+EβM) to remove the abundant heteromannan (right). The yellow lines indicate cell-wall regions where transects of fluorescence intensity were determined (see Figure 4). Scale bar = 10 μm.

Analysis of patterns of RG–I epitope detection in cell walls of the tobacco seed NME in the region indicated by the yellow star in Figure 2 showed that the epitopes were not equivalently detected across the cell walls or cell-wall domains. In sections in which the abundant heteromannan had not been enzymatically removed, the LM5 epitope was most readily detected in inner cell-wall regions adjacent to the plasma membrane, the LM6 epitope was only very weakly detected in the same regions, and the RU1 epitope was not detected at all (Figure 3). After enzymatic removal of heteromannan, the LM5 epitope was more abundantly detected across the primary cell walls, but not in the middle lamellae regions. Similarly, the LM6 epitope was also detected much more widely throughout the cell walls, but still less so in the middle lamellae regions (Figure 3). In contrast, after enzymatic removal of heteromannan, the RU1 epitope was detected throughout all middle lamellae (Figure 3).

**Figure 4:**
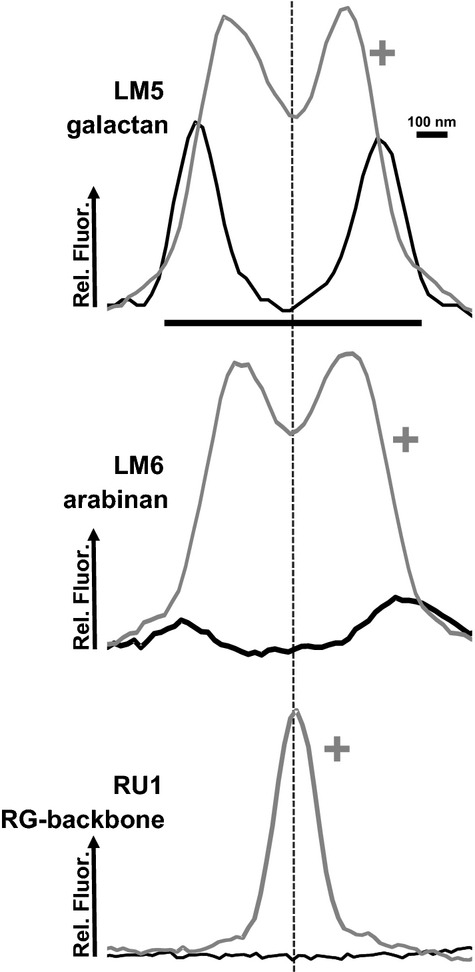
Quantification of immunofluorescence signals in transects across cell walls in adhered regions of cells in the NME of tobacco seeds. The profiles show relative fluorescence for LM5 galactan, LM6 arabinan and RU1 RG backbone epitopes in equivalent sections that had received no pre-treatment (black lines) or a pre-treatment with endo-β–mannanase (grey lines, +). Fluorescence profiles are the means of three equivalent transects taken from the regions indicated by yellow stars in Figure 2 and yellow lines in Figure 3.

To obtain insight into the relative detection of these three epitopes across adjacent cell walls (from the plasma membrane through cell wall/middle lamella/cell wall to the plasma membrane), profiles of fluorescence intensity along a transect perpendicular to the wall were generated. The profiles of relative fluorescence are shown in Figure 4, and were determined from the means of three transects in the region of the yellow bar in Figure 3. LM5 epitope fluorescence displayed two distinct peaks of fluorescence at inner cell walls with no detectable fluorescence in the central middle lamellae. After removal of heteromannan, the LM5 epitope was detected across the cell walls, but with a reduced signal in the middle lamellae region. This was similar to the LM6 profile, although the difference in relative fluorescence between the presence and absence of heteromannan was greater. In the case of the RU1 epitope, its presence in the middle lamellae was confirmed.

These observations indicate that, at the level of a single cell wall or two adhered cell walls, the three epitopes may be differentially masked by heteromannan (RU1 > LM6 > LM5). It also clearly indicates a distinctive detection profile for the RU1 epitope, which is abundant in the middle lamellae regions, compared to the LM6 and LM5 epitopes (most abundant in the adjacent cell walls).

### Approaches to determine the underlying biochemical heterogeneity of RG–I glycans using an epitope detection anion-exchange chromatography assay

In the analyses discussed above, glycan epitopes of RG–I were detected in cell walls in a single tissue using fluorescence imaging approaches. Such approaches provide no information on the connections between epitopes, although all three epitopes are proposed to be structural features of the RG–I pectic glycan. The non-uniform detection of these three epitopes at the tissue level indicates that there is no single form of RG–I, but that distinct forms or sub-populations may exist, such as arabinan-rich and galactan-poor RG–I in ME cell walls. It is of course also possible that the three epitopes may not be always connected within RG–I molecules. To address these questions, and to improve understanding of the underlying biochemical complexity of the RG–I molecules that carry these epitopes (structural features), the various antibodies were used as detection tools in a series of sandwich ELISAs and in anion-exchange chromatographic separations using cell-wall polymers solubilized from tobacco endosperm.

**Figure 5:**
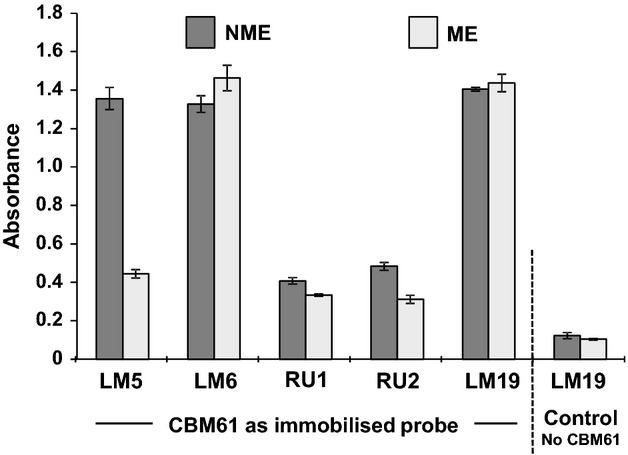
Sandwich ELISA of RG–I probe binding to extracted cell-wall polymers incubated in wells coated with 1,4–galactan-binding CBM61. Control wells without CBM61 coating were incubated with NME and ME extracts and also RG–I probes, and gave background only signals as shown for LM19. Error bars indicate the SD of three replicates.

Endosperms were isolated from 50 seeds, the ME and NME ends were excised, and the cell-wall polymers from each half were solubilized by alkaline extraction. To determine whether the RG–I epitopes were present and connected within pectic macromolecules, a sandwich ELISA was performed using CBM61, a galactan-binding carbohydrate-binding molecule (Cid *et al*., 2010), as an immobilized galactan probe to capture RG–I molecules. Alkaline-extracted glycans from the equivalent of a quarter of an endosperm per well were incubated with immobilized CBM61, and then, after washing, the CBM61/RG–I complexes were probed with LM5 galactan, LM6 arabinan, RU1 and RU2 RG backbone probes and also with the LM19 homogalacturonan probe (Verhertbruggen *et al*., 2009a). The results shown in Figure 5 indicate that the LM5, LM6 and LM19 probes bound strongly to the CBM61/RG–I complexes, indicating connection of these epitopes to the galactan domain bound by CBM61. RU1 and RU2 antibodies also bound, but to a lesser extent. The greatest differential between the NME and ME extracts was observed with the LM5 galactan antibody, which displayed a reduced signal with the ME fraction, as predicted from the *in situ* analyses. Omission of CBM61 as a coating of the microtitre plate resulted in a background signal with the antibodies, as shown for LM19 (Figure 5).

**Figure 6:**
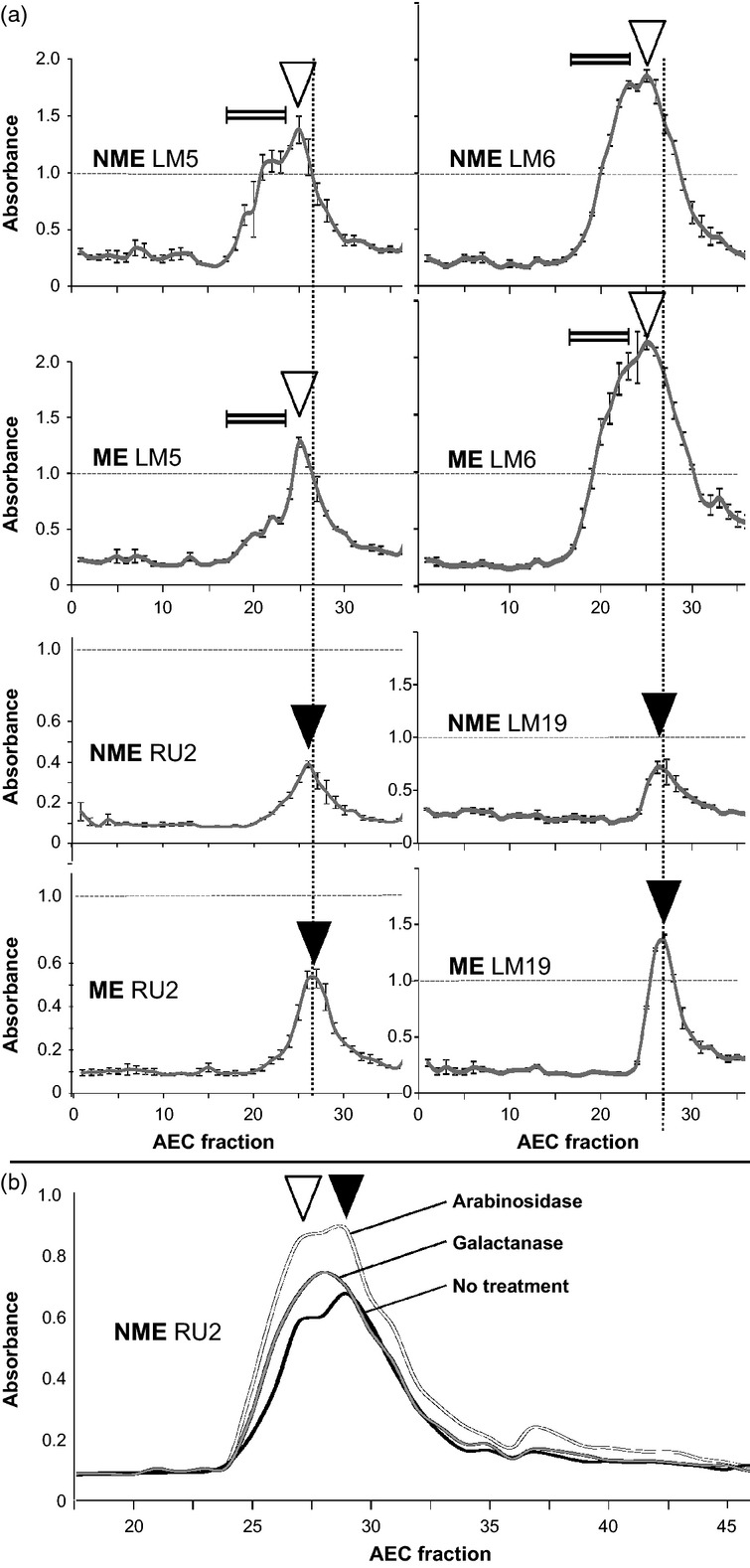
Epitope detection anion-exchange chromatography of extracts of the NME and ME halves of tobacco seed endosperms. Isolated cell-wall materials were fractionated using an anion-exchange chromatography column, and epitopes were detected in the same fractions by ELISA using LM5 galactan, LM6 arabinan, RU2 RG–I backbone and LM19 homogalacturonan (HG) monoclonal antibodies. Fractions indicated by the double-line symbol indicate the presence of early-eluting material using the LM5 and LM6 epitopes ahead of the peak of RG backbone and HG elution. Galactan-containing early-eluting peaks were more abundant in the NME than in the ME, and HG was more abundant in the ME than in the NME. Open triangles indicate coincident peaks of LM5 and LM6 epitope elution. Closed triangles indicate coincident peaks of RU2 and LM19 epitope elution. Vertical dotted lines indicate peaks of HG elution. Error bars indicate the SD of three absorbance values.

Equivalent alkali-extracted materials (approximately five endosperm halves) were injected into a low-pressure chromatography system with a 1 ml anion-exchange column. Aliquots (100 μl) of 1 ml fractions were incubated in microtitre plate wells, and ELISAs were used to generate the detection profiles shown in Figure 6(a). The LM5 epitope was detected in fractions 18–35, and equivalent peaks of recognition in terms of elution volume (indicated by open triangles) were observed in material from both the ME and NME. However, LM5 recognition of the separated NME material had more shoulders and sub-peaks in the early-eluting fractions (less acidic) (indicated by the double line Figure 6a) than the ME material. Similar profiles were obtained for the LM6 epitope, although there was less difference between the ME and NME fractions compared with the LM5 profiles. This again reflects the occurrence of the epitopes in immunocytochemical analyses. In contrast, the peak for the RU2 epitope (which consistently produced stronger responses in the epitope detection protocols than RU1), occurred slightly later than for the LM5/LM6 peak (closed triangles) and also coincided with peaks of pectic HG elution as indicated by the LM19 pectic HG epitope. It was also noted that the RU2 and HG epitopes in the NME fraction were present slightly earlier than in the ME fraction, but both peaks occurred after the peaks of LM5/LM6 elution.

These observations from both sandwich ELISA and chromatography experiments, i.e. that the three epitopes are attached to acidic molecules and that all broadly elute in the same region that overlaps with HG, suggests that some RG–I molecules are linked to HG domains. The subtle differences in peak shapes and elution times are suggestive of a range of RG–I forms, and some earlier-eluting forms have relatively reduced acidic domains. The lack of complete coincidence of elution peaks for the LM5 and LM6 epitopes with RU2 epitopes may indicate that these epitopes are attached to different molecules, or, alternatively, that, in some cases, the presence of side chains restricts access of the RU2 antibody to the backbone. Evidence for this was obtained by semi-quantitative ELISA analysis of equivalent fractions of NME material on ELISA plates using the RU2 antibody after the material had been incubated in the microtitre plate wells with a galactanase or an arabinosidase (Figure 6b). Both of these treatments to some extent increased detection of the RU2 epitope, and also showed increased recognition of earlier-eluting polymers, indicating that the RG–I polymers are possibly more heavily branched in these fractions.

## Discussion

### Extending knowledge of glycan masking in plant cell walls

Plant cell-wall immunocytochemistry has recently been enlivened by the demonstration that, in some cases, abundant pectic HG obscures or masks antibody or protein access to co-extensive xyloglucan and heteromannan epitopes (Marcus *et al*., 2008, 2010; Davies *et al*., 2012). Here we demonstrate that, in tobacco seed endosperm, where heteromannan is at least three times more abundant than cellulose (Lee *et al*., 2012), it may partially and differentially mask access to the LM6 arabinan epitope and also blocks access to the RU1 RG–I backbone epitope. This work confirms that RG–I epitopes may be masked (Yu *et al*., 2011), and extends knowledge of cell-wall masking in that glycans other than pectic HG may block access/detection of cell wall glycans, that RG–I epitopes may be differentially masked by a glycan, and that the extent of masking may be regulated within a tissue. The precise mechanism and biological significance of glycan masking within cell-wall architectures remains to be elucidated. The differential masking of antibody access to the arabinan epitope reported here appears to relate to the role of the endosperm in seed germination. The cell walls of the micropylar end of the tobacco seed endosperm, with their differential cell-wall architecture, are specifically degraded during germination to allow radicle emergence (Lee *et al*., 2012). The enhanced access of the LM6 antibody to the ME cell walls may be hypothesized to reflect enhanced cell-wall porosity, allowing increased access of cell-wall remodelling enzymes to this region of the endosperm.

### Multi-scale spatial heterogeneity of RG–I epitopes

The LM5 galactan and LM6 arabinan antibody probes have been widely used to demonstrate the developmental dynamics of galactan and arabinan epitopes during plant growth and in relation to cell differentiation in organ systems and at meristems (e.g. Willats *et al*., 1999). The current hypothesis is that galactan- and arabinan-rich forms of RG–I, present in cell walls, lead to distinct mechanical properties such as stiffness or flexibility. No consistent model for developmental regulation and generation of properties across shoot and root meristems or across species has yet emerged. Here we report differential detection of the LM5 and LM6 epitopes within a single tissue, and propose that a galactan- and arabinan-rich RG–I is present in the NME, in contrast to the mostly arabinan-rich RG–I in the cell walls of the ME. This probably reflects the required mechanical properties and increased flexibility/cell-wall softness required for radicle emergence through the endosperm in the ME during germination (Lee *et al*., 2012). The genetic and physiological factors that result in modulation of RG–I structure and the mechanistic aspects of how it modulates cell-wall properties during endosperm development are not known, but appear to be an important aspect of the development of cell-wall properties and tissue and organ mechanics in general.

In this study, we used the LM5 and LM6 probes in combination with the RU1/RU2 probes for the RG–I backbone, and the observation that is most surprising is the heterogeneity of detection profiles for the three epitopes at the cell-wall level. The detection of the RU1 epitope in the middle lamellae and the LM5 and LM6 epitopes in adjacent primary cell-wall regions leads to questions concerning the spatial configuration and diversity of RG–I molecules within cell walls and intercellular matrices. It has previously been suggested that RG–I occurs in the middle lamellae (Moore *et al*., 1986), and also that the orientation of RG–I in the cell walls involves its backbones being parallel to the middle lamellae with its side chains at right angles to this (Vincken *et al*., 2003). Enzymatic disruption of RG–I backbones in potato tubers (*Solanum tuberosum*) resulted in re-location of the LM5 galactan epitope to the middle lamellae regions (Oomen *et al*., 2002). Does detection of the RU1 epitope in the middle lamellae indicate the presence of RG–I backbones in the middle lamellae with connected side chains oriented within adjacent cell walls? An alternative explanation is that there are a range of RG–I molecules in the middle lamellae and adjacent primary cell walls. In this scenario, RG–I polymers with a low density of side chains predominate in the middle lamellae, whereas RG–I molecules with a high abundance of arabinan or galactan or both and a backbone that is obscured by the abundance of side chains predominate in the cell walls. However, a range of enzymatic treatments aimed at galactan and arabinan degradation did not uncover the RU1 epitope in cell walls. It is also possible that there is differential masking of epitopes across cell walls and middle lamellae by cell-wall polymers that are not examined here. Another point to consider here is that the LM6 epitope has also been found in some cases to be carried by arabinogalactan proteins (Lee *et al*. 2005), and this may be the case for the endosperm cell walls. Arabinogalactan proteins are also potentially linked to RG–I domains (Caffall and Mohnen, 2009; Yapo, 2011b).

### Use of epitope detection chromatography and equivalent methodologies for cell-wall analyses

Much remains to be revealed concerning RG–I biochemistry and its integration within pectic supramolecules and wider cell-wall architectures. It is well established that RG–I encompasses a set of glycans of considerable heterogeneity (Yapo, 2011b). An adjunct to understanding the complex regulation and dynamics of RG–I epitopes in development of tissue and organ properties is knowledge of the underlying biochemistry of RG–I polymers. An important factor in establishing the metabolic framework for RG–I synthesis, modification and function is understanding of the occurrence and diversity of RG–I sub-populations. Combining use of the specific and sensitive antibody probes as detection tools in small-scale chromatographic separations reveals RG–I complexity and distributions. The occurrence of coincident peaks of elution of the LM5 and LM6 epitopes from the anion-exchange column earlier than the separate coincident peaks of the RU2 and LM19 HG epitopes is suggestive of a range of RG–I glycans with varying side-chain densities, and thus supports the view that the *in situ* patterns of detection reflect gradients of RG–I occurrence within cell-wall matrices. This is a powerful approach that also has the potential to provide details of the connections between RG–I domains and other cell-wall polymers within the pectic supramolecules, and also with other polymers such as attached xyloglucan domains, or their presence of RG-I domains proteoglycans that have recently been characterized (Tan *et al*., 2013). It is also important to note the high sensitivity of the epitope detection anion-exchange chromatography methodology, which allows analyses of cell-wall materials at levels at which sugar analyses are not possible. Extension of such approaches to include orthogonal separation techniques such as affinity separations is essential to identify and characterize specific RG–I forms and how they are integrated into specific cell walls or cell-wall regions and how they modulate cell-wall properties.

In summary, by combining use of the LM5, LM6 and RU1/RU2 probes, we have demonstrated that three structural features of RG–I may be differentially detected within a single tissue and within individual cell walls/intercellular matrices, and moreover that they may be differentially masked by an abundant non-cellulosic glycan in both these contexts. The aim of the epitope detection chromatography described here was to determine the underlying biochemistry of RG–I, and is indicative of high levels of RG–I heterogeneity and diversity within the cell walls of one cell type. This approach suggests a way to dissect populations of RG–I glycans and thus obtain a more nuanced understanding of the *in situ* dynamics of spatially mapped RG–I epitopes.

## Experimental procedures

### Plant materials

Tobacco (*Nicotiana tabacum* ‘Havana 425’) seeds were germinated in 10 cm^2^ Petri dishes containing 6 ml of one-tenth strength Murashige and Skoog basal medium (Murashige and Skoog, 1962) without hormones or vitamins (Duchefa, http://www.duchefa-biochemie.nl), adjusted to pH 7.0, on two layers of Whatman (http://www.whatman.com/) 3MM filter paper under continuous light at 24°C as described previously (Lee *et al*., 2012).

### Monoclonal antibodies

Three rat monoclonal antibodies (LM6 arabinan, Willats *et al*. 1998; LM5 galactan, Jones *et al*., 1997; LM19 homogalacturonan, Verhertbruggen *et al*., 2009a) and two mouse monoclonal antibodies (INRA-RU1 and INRA-RU2, Ralet *et al*., 2010) were used in this study.

### Immunocytochemistry, fluorescence imaging and fluorescence profiling

Procedures for fixation, resin-embedding and sectioning of tobacco seeds were performed as described previously (Lee *et al*., 2012). Immunocytochemistry was performed on 0.5 μm thick sections before fluorescence imaging as described previously (Lee *et al*. 2012). Quantitative analysis of fluorescence intensities across cell walls in response to antibody binding was performed using ImageJ (http://rsbweb.nih.gov/ij/) as follows: the line tool was used to generate transects at right angles to the identified region of adhered cell walls, associated fluorescence intensities were analysed using the plot profile feature, and the data were averaged for three transect lines per antibody from the same cell-wall region and adjusted to remove background fluorescence before plotting.

### Sandwich ELISA analysis of RG–I/HG epitope connections and epitope detection anion-exchange chromatography of RG–I and HG epitopes

The endosperms of 50 tobacco seeds that had been imbibed for 3 h were dissected into micropylar (ME) and non-micropylar (NME) halves. The samples were snap-frozen, and then macerated/extracted in 300 μl of 4 M KOH/1% w/v NaBH_4_ using a TissueLyser (Qiagen, http://www.qiagen.com/), and neutralized with 80% v/v acetic acid to give a total volume of 380 μl.

Aliquots of NME and ME extracts were used in sandwich ELISA analyses. Microtitre plates (Sigma-Aldrich, http://www.sigmaaldrich.com/) were coated overnight with galactan-binding CBM61 protein (Cid *et al*., 2010) at 1 μg ml^−1^ in PBS. After blocking with 3% w/v milk powder in 1 × PBS (137 mm NaCl, 2.7 mm KCl, 10 mm Na_2_HPO_4_, 2 mm KH_2_PO_4_) (MP/PBS) for 1 h, aliquots of the cell-wall extracts (equivalent to a quarter endosperm per well) were incubated in MP/PBS for 2 h. The plates were washed with water nine times for 30 s at room temperature 20°C and shaken dry. After washing, any bound materials were probed with the RG–I antibodies at 100 μl per well of a 1 in 10 dilution in MP/PBS, and incubated for 1 h. After a further similar washing step, secondary antibodies (anti-rat or horseradish peroxidase-conjugated anti-mouse IgG) were added at 100 μl per well of a 1 in 1000 dilution, and incubated at room temperature for 1 h. After a further washing step, the presence of bound antibody was revealed by addition of 150 μl substrate per well (0.1 m sodium acetate buffer pH 6, 1% w/v tetramethyl benzidene, 0.006% v/v H_2_O_2_). The reaction was stopped by addition of 40 μl of 2 M H_2_SO_4_ per well, and the absorbance was read at 450 nm on a microtitre plate reader.

For anion-exchange analyses, 50 μl aliquots of NME and ME alkaline extracts were diluted in 2.5 ml water, and run through an anion-exchange column (1 ml HiTrap ANX FF, GE Healthcare, http://www3.gehealthcare.com/) with 20 mm sodium acetate buffer, pH 4.5, from 0 to 17 min, with a step change to 50 mM sodium acetate buffer, pH 4.5, at 17 min, then a linear gradient from 0 to 100% 0.6 m NaCl from 17 to 48 min, followed by 8 min of 50 mm acetate with 0.6 m NaCl. Forty-eight 1 ml fractions were collected. Between injections, the column was washed with 5 ml of 0.1 m NaOH. The column was then equilibrated, and the injection loop was flushed with 10 ml of 20 mm acetate buffer prior to the next run. Collected fractions were adjusted to pH 9 using 40 μl of 1 m Na_2_CO_3_, and 100 μl aliquots were incubated in microtitre plate wells overnight at 4°C. The plates were washed vigorously with tap water and shaken dry, and assayed for binding of RG–I probes as described above.

In some cases, fractions immobilized on microtitre plates were treated with enzymes prior to the antibody reactions. This involved use of an α–l–arabinofuranosidase (*Aspergillus niger*, 10 μg/ml, Megazyme, http://www.megazyme.com/) or an endo-1,4–β–d–galactanase (*A. niger*, 10 μg ml^−1^, Megazyme) in 50 mm acetate buffer, pH 4.5, and incubation for 4 h at 37°C. The microtitre plates were then washed and processed as described above.
